# Perceptions of Medical Residents About Sleep Disorders and Sleep Medicine in Qassim Province, Saudi Arabia

**DOI:** 10.7759/cureus.78874

**Published:** 2025-02-11

**Authors:** Turki I Aloraini, Chandra Sekhar

**Affiliations:** 1 Family Medicine, Family Medicine Academy, Qassim Health Cluster, Buraidah, SAU

**Keywords:** challenges, prevalence of sleep disorder, qassim residents, risk factors, saudi arabia, suggestions

## Abstract

Background

Sleep disorders have increased drastically over the past few decades due to lifestyle, environmental, and geographical factors and individual high-level financial goals and requirements. The study aims to identify the prevalence of sleep disorders among Qassim residents and to determine the risk factors associated with sleep disorders.

Methods

A cross-sectional study was conducted among 150 Saudi Board residents of different postgraduate programs in Qassim Province using a self-administered questionnaire through Google Forms (Google, Inc., Mountain View, CA), and 105 participants responded. Data was analyzed using the SPSS version 21.0 (IBM Corp., Armonk, NY) and necessary statistical tests; the chi-square test was applied for sleep disorder symptoms and risk factors, with the prevalence of sleep disorder as a category.

Results

In our study, 43.8% (n=46) were men; the mean age and standard deviation in the study population was 28.30±2.77 years. Approximately 98.1% (n=103) of residents were aware of sleep disorders. About 82.9% (n=87) of residents were aware of breathing difficulty for a sleep disorder. Regarding symptoms of obstructive sleep apnea (OSA), hypersomnia and hyposomnia prevalence were 40% (n=42) and 41.9% (n=44), respectively. Concerning challenges, about 98.5% (n=68) believed that training is required to treat sleep disorders. About suggestions, 22.7% (n=10) stated that lifestyle modifications are required, and 20.5% (n=9) mentioned the establishment of sleep clinics. There was a statistically significant association observed between sleep disorder prevalence with sleepwalking, hypersomnia, and hyposomnia (P<0.05).

Conclusions

Based on the study findings, the Qassim Board residents' awareness of sleep disorders is excellent. More than two-fifths of residents (40% and 41.9%) had hypersomnia and hyposomnia symptoms, respectively. Most residents (98.5%) mentioned that training is required to treat sleep disorders as a challenge and suggested lifestyle modifications and sleep clinics.

## Introduction

Sleep disorders are a significant public health concern, with an estimated prevalence of up to 35% in the general population [1]. These disorders can have far-reaching consequences, including impaired cognitive function, decreased quality of life, and increased risk of chronic diseases such as obesity, diabetes, and cardiovascular disorders [2]. A study from the United States of America (USA) reported that postmenopausal veteran women have a high risk for insomnia [3]. Despite their prevalence and impact, sleep disorders often go undiagnosed and untreated, partly due to a lack of awareness and knowledge among healthcare professionals [4].

Saudi Arabia is the fifth largest country in Asia, with a population of 32.2 million, according to the General Authority for Statistics, Saudi Arabia 2022 census (https://www.spa.gov.sa/w1911463). Concerning the population needs, there is enormous potential to improve sleep medicine facilities [5] in the country, comparatively to other developed countries such as the USA, the United Kingdom, Canada, Australia, and Spain [6].

The practice of sleep medicine in Saudi Arabia started in the late 1990s; also, based on the current evidence of practice, sleep disorder prevalence is increasing globally, including in Saudi Arabia. Based on the two studies conducted in Saudi Arabia, three out of every 10 middle-aged Saudi men and four out of every 10 Saudi women are at risk of developing sleep disorders, including obstructive sleep apnea (OSA) [7,8]. Hence, our services were proportionally expanded to be implemented from the primary healthcare center (PHCC) level to achieve optimum utilization of services for the population.

In Saudi Arabia, studies have reported a high prevalence of sleep disorders, with rates ranging from 33.8% to 57.5% in different regions [9]. During COVID-19, in Aseer region public health facilities, 85.1% of Saudi Commission for Health Specialties (SCFHS) residents had poor sleep quality [10]. In a Singaporean study, individuals reported more sleep, varying by employment, personal behavior, and ethnicity [11]. However, there is a lack of research exploring the knowledge and attitudes of medical professionals, particularly residents, toward sleep disorders and sleep medicine in the country [12].

Adequate knowledge and training in sleep medicine are crucial for medical residents, as they are often the first point of contact for patients with sleep-related issues. Several studies have highlighted the need for improving sleep education in medical curricula worldwide [13]. In the USA, a survey found that only 27% of medical residents felt adequately prepared to manage sleep disorders [14].

In Saudi Arabia, limited studies have explored medical residents' perceptions of sleep disorders and sleep medicine. A study conducted in Riyadh found that only 26.5% of residents had received formal training in sleep medicine, and their overall knowledge was suboptimal [15]. Another study at Qassim University among medical students reported that the knowledge of sleep medicine was low [16].

SCFHS made a compulsion to start a sleep medicine program at the national level. The program recommended that trainees must be confident enough to diagnose and manage different sleep disorders [17]. This study aims to assess the perceptions of medical residents in Qassim Province, Saudi Arabia, toward sleep disorders and sleep medicine promotional measures. The study aims to find the prevalence, risk factors, and challenges of sleep disorders and their associations and suggestions to improve sleep disorder management.

## Materials and methods

Study setting, target population, and design

The current study's target population was the postgraduate medical residents in the Qassim Board programs of SCFHS. A cross-sectional study was conducted among all the resident doctors of Qassim over a period of one year.

Sample size and sampling

Using the Open Source Epidemiologic Statistics for Public Health sample size calculator (https://www.openepi.com/Menu/OE_Menu.htm), the following parameters were applied to estimate the sample size. The number of residents in Qassim Province in all Saudi Board programs was approximately 150 based on the time of study protocol. The estimated prevalence of sleep disorder was 50%, with 95% confidence limits and design effect one. Based on the above prerequisites, the estimated sample was 120 at a 95% confidence interval (CI).

All the residents belonging to Qassim medical residency programs meeting our inclusion criteria were recruited as a sample. Hence, no specific sampling method was applied in this study, just like a census enumeration.

Inclusion criteria and exclusion criteria

Postgraduate residents working at different primary healthcare centers and hospitals of Qassim of both genders have shown interest in the research. Residents with any psychological disorders were excluded. 

Data collection tool

Data was collected through a semi-structured questionnaire, which consists of primarily closed-ended questions and some open-ended questions. The data collection tool was developed from previous studies [5,10,17]. After developing the questionnaire, it was initially reviewed with family medicine consultants and research-experienced faculty. Their suggestions were incorporated. The questionnaire's validity was assessed by examining its face validity, content validity, reliability, and linguistic issues. Then, a pilot study was planned to refine it.

The questionnaire consists of five sections (Appendices). The first section deals with the participants' demographic characteristics (age, gender, resident level, name of the board program, and financial problem), and the second section denotes awareness about sleep disorders (excessive daytime sleep, loud snoring, breathing difficulty, working during night, morning headache, the lack of concentration, mood changes, and sleep disturbances). The third section states the self-reported risk factors for sleep disorders (physical activity, the duration of the activity, sleepwalking, nightmares, hypersomnia, hyposomnia, smoking, and coffee drinking); the fourth section denotes challenges mentioned in closed-ended questions created as yes/no options (training required, confidence in the provision of treatment, the interest of resident, the availability of medication, compliance with medication, administrative support, colleague support, and facility provision). Lastly, the fifth section mentions suggestions to promote sleep medicine treatment modalities. This suggestion question is not provided as a mandatory option in a Google Form. Out of 105 residents, we received 44 responses to suggestion questions. All these 44 responses were further classified into eight themes (establish sleep clinics, workshops, lifestyle modifications, referral, more training, screening program at a PHCC, awareness, and health education).

A self-administered questionnaire was distributed to the participants using an online platform via Google Forms (Google, Inc., Mountain View, CA) (through WhatsApp social media {Meta Platforms, Menlo Park, CA}). Each participant communicated in a direct message, as well as in groups. During the data collection, in the case of direct messages, oral consent was taken, and informed consent was also attached on the first page of the Google Form; once they agreed, the questionnaire would appear. In the case of group messages, it is not possible to give oral consent but inferred informed consent through the first page of the Google Form.

Ethical considerations

After the research proposal was completed, it was submitted to the Qassim Regional Ethics Committee with approval number 607/45/14986 dated May 15, 2024. Informed consent was obtained from every participant, both oral and agreed consent, on a Google Form. The confidentiality of the individual information was maintained, and data was not shared with any private or public agencies.

Pilot study

After obtaining the ethical committee's approval, a pilot study was conducted among 15 resident doctors to determine the field's technical feasibility and ensure the questions' presentation and order. The pilot study sample was not included in the main study sample. After the pilot study, we did not change our questionnaire, and that situation did not arrive.

Statistical analysis

A comprehensive nature of data entry, management, and statistical analysis (using SPSS version 21.0 {IBM Corp., Armonk, NY}), including descriptive and inferential statistics, was used to draw the inferences. Initially, descriptive analysis was conducted on demographic characteristics. Also, certain variables, such as the comparison between the sleep disorder risk factors concerning the prevalence of sleep disorder, were done to find the association. Means and standard deviations were calculated for the continuous variables in the study. An independent t-test was applied to the continuous variable body mass index (BMI) and the category of sleep disorder. For all the categorical variables, the chi-square test was applied. The level of statistical significance will be taken as the probability (P) value is less than or equal to 0.05.

## Results

Approximately 105 Saudi Board residents have participated in the current study. We distributed our questionnaire through a Google link to approximately 150 residents of Qassim Province who work at the different PHCCs and hospitals. The response rate in the study population was 70% (105/150 approximately). The mean age in the study population was about 28.30±2.77 years (range is 12 years). Seventy-five percent of the study participants were below 29 years of age.

About 43.8% (n=46) were men, and 33.3% (n=35) were Saudi Board R3 residents in the Qassim region. In the study population, about 55.2% (n=58) were from the family medicine program, 12.4% (n=13) from general surgery, 8.6% (n=9) from internal medicine, 9.5% (n=10) from pediatrics, and 4.8% (n=5) each from psychiatry, dermatology, and others. Only 23.8% (n=25) had financial problems. The mean BMI in the study population was 25.36±4.77 kg/m^2^. Among the sleep disorder residents, the mean BMI was 26.53±5.01 kg/m^2^, and among non-sleep disorder residents, the mean BMI was 24.55±4.45 kg/m^2^; this association was statistically significant (P<0.05) (analyzed and not mentioned in the following table). About 38.1% (n=40) were not practicing physical activity. Among the physical activity-practicing study population, about 41% (n=43) of residents practiced physical activity for >30 minutes/per day. Approximately 83.8% (n=88) of residents were completing their academic activity regularly (Table 1).

**Table 1:** Demographic characteristics of the Saudi Board residents of Qassim Province. n, number; %, percentage; BMI, body mass index; SD, standard deviation

Nationality	Number of participants (n)	Percentage (%)
Age±SD	28.30±2.77 years	
Gender		
Male	46	43.8
Female	59	56.2
Resident level		
R1	29	27.6
R2	30	28.6
R3	35	33.3
R4	7	6.7
R5	4	3.8
Any financial problem		
Yes	25	23.8
No	80	76.2
BMI±SD	25.36±4.77	
Physical activity		
Yes	65	61.9
No	40	38.1
Duration of physical activity		
<30 minutes/day	37	35.2
>30 minutes/day	43	41.0
Completion of academic activities regularly		
Yes	88	83.8
No	17	16.2

Table 2 shows that about 98.1% (n=103) of residents are aware of sleep disorders. Approximately 89.5% (n=94) opined that sleep disturbance was the predominant symptom of sleep disorder, followed by excessive daytime sleep, which was about 85.7% (n=90). About 80% (n=84) of residents are aware of loud snoring as a cause of sleep disorder. Nearly 82.9% (n=87) of residents are aware of breathing difficulty for a sleep disorder.

**Table 2:** Awareness regarding risk factors of sleep disorders in the study population. n, number; %, percentage; OSA, obstructive sleep apnea

Variables	Yes (%)	No (%)	Total (%)
Heard about OSA	103 (98.1)	2 (1.9)	105 (100)
Excessive daytime sleep	90 (85.7)	15 (14.3)	105 (100)
Loud snoring	84 (80)	21 (20)	105 (100)
Breathing difficulty	87 (82.9)	18 (17.1)	105 (100)
Walking during the night	47 (44.8)	58 (55.2)	105 (100)
Morning headache	80 (76.2)	25 (23.8)	105 (100)
Lack of concentration	86 (81.9)	19 (18.1)	105 (100)
Depression	86 (81.9)	18 (18.1)	105 (100)
Sleep disturbance	94 (89.5)	11 (10.5)	105 (100)

Table 3 depicts that in the study population, among the observed risk factors for sleep disorders, smoking has the lowest prevalence at 4.8% (n=5), and coffee drinking has the highest at 74.3% (n=78). Hypersomnia and hyposomnia prevalence were 40% (n=42) and 41.9% (n=44), respectively. About 29.5% (n=31) of residents were aware that nightmare is a risk factor for sleep disorders.

**Table 3:** Risk factors of sleep disorder in the study population. n, number; %, percentage

Risk factors	Yes (%)	No (%)	Total (%)
Smoking	5 (4.8)	100 (95.2)	105 (100)
Coffee drinking	78 (74.3)	27 (25.7)	105 (100)
Sleepwalking	8 (7.6)	97 (92.4)	105 (100)
Nightmares	31 (29.5)	74 (70.5)	105 (100)
Hypersomnia	42 (40)	63 (60)	105 (100)
Hyposomnia	44 (41.9)	61 (58.1)	105 (100)

Table 4 reveals that about 65.7% (n=69) mentioned that treating sleep disorders was challenging. The highest number of residents, 98.5% (n=68), gave an opinion that training is required to treat sleep disorders, and 69.5% (n=48) of residents stated that facility provision is required to treat sleep disorders. About 59.4% (n=41) of residents opined that they were confident in the treatment of sleep disorder, and, 60.8% (n=42) mentioned that they needed administrative support.

**Table 4:** Challenges in treating sleep disorders in the study population. n, number; %, percentage

Variables	Yes (%)	No (%)	Total (%)
Challenges to treatment	69 (65.7)	36 (34.3)	105 (100)
Training required	68 (98.5)	1 (1.5)	69 (100)
Confident in treatment	41 (59.4)	28 (40.6)	69 (100)
Interest to treat	39 (56.5)	30 (43.5)	69 (100)
Administration support	42 (60.8)	27 (39.2)	69 (100)
Colleague support	44 (63.7)	25 (36.3)	69 (100)
Facility provision	48 (69.5)	21 (30.5)	69 (100)

**Figure 1:**
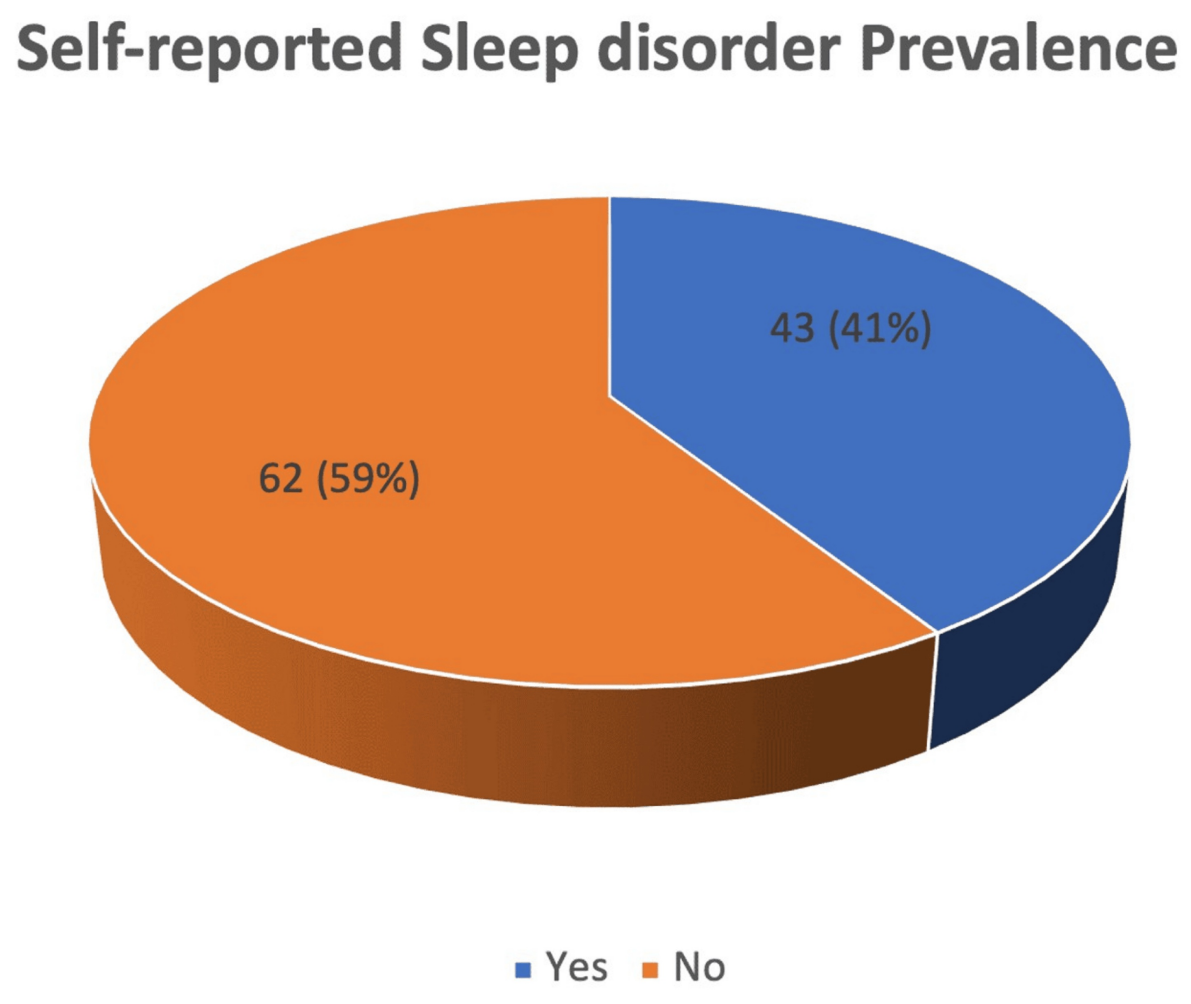
Prevalence of sleep disorder in the study population. About 41% (n=43) self-reported the prevalence of sleep disorder in the study population.

**Figure 2:**
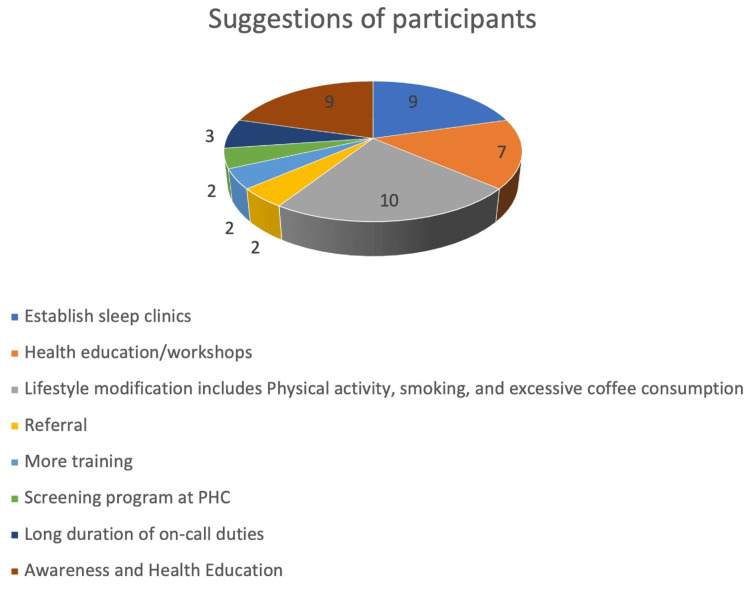
Suggestions for improving sleep disorder management in the study population. PHC: primary healthcare

In our study, approximately 22.7% (n=10) stated that lifestyle modifications are required, and 20.5% (n=9) mentioned the establishment of sleep clinics and awareness and health education about sleep disorder management. Out of 105 residents, only 37 residents gave their suggestions to improve sleep disorder management. Some gave more than one response. Hence, the total number of responses is 44. Nearly 20.5% (n=9) of residents stated that establishing a sleep clinic is a suggestion to improve sleep disorder management. Approximately 15.9% (n=7) of residents mentioned that health education workshops are needed to improve sleep disorder management (Figures 1, 2).

Among the sleepwalking symptom presenters, a prevalence of about 87.5% (n=7) of sleep disorder was observed; in no-sleepwalking residents, the prevalence of sleep disorder was 12.5% (n=1); this association was statistically significant (P<0.05). In hypersomnia residents, 64.3% (n=27) had a sleep disorder, and for no-hypersomnia residents, the prevalence was 25.4% (n=16), and a statistically significant association was found (P<0.05). Among the nightmare residents, the prevalence of sleep disorder was 64.5% (n=20), and among non-nightmare residents, the prevalence of sleep disorder was 31.1% (n=23); this association was statistically significant (P<0.05) (Table 5).

**Table 5:** Risk factors associated with sleep disorder prevalence in the study population. *Statistically significant. X^2^, chi-square test; P, probability; OR, odds ratio; CI, confidence interval

Risk factors	Categories	Sleep disorder		X^2^, P value, OR, and CI
		Yes	No	X^2^: 7.759; P=0.005*; OR=11.86; CI: 1.402-100.35
Sleepwalking	Yes	7 (87.5%)	1 (12.5%)	
	No	36 (37.1%)	61 (62.9%)	
Nightmares	Yes	20 (64.5%)	11 (35.5%)	X^2^:10.10; P=0.001*; OR=4.03; CI: 1.664-9.771
	No	23 (31.1%)	51 (68.9%)	
Hypersomnia	Yes	27 (64.3%)	15 (35.7%)	X^2^:15.76; P=0.0001*; OR=5.28; CI: 2.263-12.354
	No	16 (25.4%)	47 (74.6%)	
Hyposomnia	Yes	31 (70.5%)	13 (29.5%)	X^2^:27.26; P=0.0001*; OR=9.73; CI: 3.941-24.055
	No	12 (19.7%)	49 (80.3%)	
Smoking	Yes	2 (40%)	3 (60%)	X^2^: 1.52; P=0.678; OR=0.959; CI: 0.153-5.999
	No	39 (40.6%)	57 (59.4%)	
Coffee drinking	Yes	26 (33.3%)	52 (66.7%)	X^2^:7.282; P=0.007*; OR=0.294; CI: 0.118-0.732
	No	17 (63%)	10 (37%)	

Among the excessive daytime sleep residents, there was a prevalence of 45.6% (n=41) of sleep disorder, whereas for non-excessive daytime sleep residents, the prevalence of sleep disorder was 13.3% (n=2); this association was statistically significant (P<0.05). Other symptoms of residents with sleep disorder, such as loud snoring, breathing difficulty, walking at night, morning headache, lack of concentration, depression mood changes, and sleep disturbance, were not statistically significant (P>0.05) (Table 6).

**Table 6:** OSA symptoms associated with sleep disorder in the study population. *Statistically significant. X^2^, chi-square test; P, probability; OR, odds ratio; CI, confidence interval; OSA, obstructive sleep apnea

Symptoms	Categories	Sleep disorder		X^2^, P value, OR, and CI
		Yes	No	X^2^: 5.52; P=0.019*; OR=5.43; CI: 1.160-25.50
Excessive daytime sleep	Yes	41 (45.6%)	49 (54.4%)	
	No	2 (13.3%)	13 (86.7%)	
Loud snoring	Yes	33 (39.3%)	51 (60.7%)	X^2^: 0.482; P=0.487; OR=0.71; CI: 0.272-1.862
	No	10 (47.6%)	11 (52.4%)	
Breathing difficulty	Yes	32 (36.8%)	55 (63.2%)	X^2^: 3.65; P=0.056; OR=0.370; CI: 0.130-1.051
	No	11 (61.1%)	7 (38.9%)	
Walking at night	Yes	23 (48.9%)	24 (51.1%)	X^2^: 2.243; P=0.134; OR=1.821; CI: 0.828-4.002
	No	20 (34.5%)	38 (65.5%)	
Morning headache	Yes	33 (41.3%)	47 (58.8%)	X^2^: 0.012; P=0.912; OR=1.053; CI: 0.422-2.631
	No	10 (40%)	15 (60%)	
Lack of concentration	Yes	35 (40.7%)	51 (59.3%)	X^2^: 0.013; P=0.910; OR=0.944; CI: 0.345-2.584
	No	8 (42.1%)	11 (57.9%)	
Mood changes such as depression	Yes	36 (41.9%)	50 (58.1%)	X^2^: 0.162; P=0.687; OR=1.234; CI: 0.442-3.443
	No	7 (36.8%)	12 (63.2%)	
Sleep disturbance	Yes	39 (41.5%)	55 (58.5%)	X^2^: 0.107; P=0.744; OR=1.241; CI: 0.340-4.531
	No	4 (36.4%)	7 (63.6%)	

## Discussion

This study's results provide some updates on the perceptions of Qassim residents about sleep medicine prevalence, risk factors, challenges, and suggestions. In our study, about 41% self-reported the prevalence of sleep disorders in the study population. Our study observation was almost the same as a study from Saudi Arabia's general population; the prevalence of sleep disorders was found to be 37.6% [18]. Our study's low prevalence of sleep disorders could be due to the fact that most participants are from the family medicine program; often, these residents do not have night shifts (except in some rotations).

Some studies noted a high prevalence of sleep disorders among undergraduate and postgraduate students. A study conducted among medical students in Makkah stated that 73.8% complained of at least one sleep disorder [19]. A study in Riyadh among Saudi Arabian medical residents showed a very high prevalence of having poor sleep quality at 86.3%. This high prevalence could be due to on-call schedules and night shifts; it is a part of training [20], and among Brazilian psychiatry residents, it was 59.3% [21].

In our current study, the mean BMI in the medical residents' study population was 25.36±4.77 kg/m^2^. In a study done in Jordan, the mean BMI was 24.2±4.4 kg/m^2^ [22]. The high BMI and standard deviation noticed in a study among the sleep disorder population was 30.9±8.8 kg/m^2^ [23]. A systematic study also reported a significant association between high body mass index (>25 kg/m^2^) and sleep disorder (odds ratio {OR}: 1.33; CI: 1.16-1.51) [24]. About 80% (n=84) of residents are aware of loud snoring as a cause of sleep disorder. Snoring is a common symptom, a little more prevalent among men than women, and it is the most common symptom observed in sleep disorder patients at 70%-95% [23,25].

Residents opined that coffee drinking, 74.3% (n=78), was observed as the greatest risk factor for sleep disorder in the study. A study from Jizan found that 85.2% of students consumed coffee; 54.5% of the general population stated that coffee caused insomnia, and this is a significant observation (P<0.01). In fact, coffee consumption is a common practice worldwide among medical residents, and also, the quantity and frequency of coffee consumption are related to the causation of sleep disorders [26].

In our study, hypersomnia and hyposomnia prevalence were 40% (n=42) and 41.9% (n=44), respectively. A study from Jordan stated that hypersomnia was 23.9% and hyposomnia was 18.8% [22]. In hypersomnia residents, 64.3% had a sleep disorder, compared to 25.4% in those without hypersomnia, showing a significant association (P<0.05). Other studies from different countries also noted that hypersomnia is one of the risk factors for sleep disorder and stated that addressing the underlying cause of sleep disruption, chronic sleep deprivation, usually improves sleepiness [27,28].

About 29.5% (n=31) of residents were aware that nightmare is a risk factor for sleep disorders in our study. A Jordanian study stated that nightmare prevalence was 5.6% [22]. The prevalence of nightmares among sleep disorder patients, such as depressive patients, was 28.4%, which is a similar observation to our current study [29]. Most of the other studies also stated that nightmares are also associated with age among children and adolescent groups [30,31].

In the current study, about 65.7% (n=69) mentioned that treating sleep disorders was challenging. A study from Riyadh, an expert committee for sleep medicine, stated that to become competent residents, assessing, training, diagnosing, and managing sleep disorders are required [17]. In the present study, approximately 15.9% (n=7) of residents mentioned that health education workshops are needed to improve sleep disorder management. A study from China among undergraduate students also stated that universities should encourage and conduct a good number of health education sessions to minimize the prevalence of sleep disorders [32].

In our study, the participants mentioned that 22.7% (n=10) of residents require lifestyle modifications. In contrast, the study from China stated that lifestyle modifications were significantly (OR: 1.56; 95% CI: 1.46-1.68) associated with sleep health/disorder, and about 17.4% of the general population have unhealthy lifestyles [33]. In this study, about 20.5% (n=9) of residents stated that establishing a sleep clinic is a suggestion to improve sleep disorder management. A study from the USA also stated that sleep disorders can increase morbidity and healthcare costs and use. Family physicians play a vital role; if they manage the cases of sleep disorders at a PHCC, they will provide better outcomes in terms of the reduction of burden in the country [34,35]. Research in clinical practice, policies, and future research imply that improving the capability of healthcare providers to diagnose and treat sleep disorders, lifestyle modification strategies, and daytime functioning among residents somehow reduce hypersomnia and hyposomnia. The cost-effectiveness analyses of sleep medicine training and the establishment of sleep clinics at PHCC were done to understand these initiatives' financial implications and benefits on the healthcare system. In future studies, more participants from many postgraduate medical board programs must provide a comprehensive picture of sleep disorders and sleep medicine.

Some of the limitations observed in our study are that the study is a self-administered questionnaire, it had some misunderstanding of questions at the resident's level (participants), and very few people contacted the principal investigator about the misunderstanding of questions, despite all efforts from the principal investigator. Some variables, such as coffee consumption and quantity of coffee, were not included. The socially desirable bias cannot be prevented. In our study, the majority of postgraduate program residents included in this study were family medicine residents, and the generalizability of the study findings for all board residents is not advisable. In future studies, more participants from many postgraduate medical board programs will provide a comprehensive picture of sleep disorders and sleep medicine.

## Conclusions

In conclusion, in the study, Qassim Board residents had excellent awareness of sleep disorders; more than two-fifths of residents had hypersomnia and hyposomnia as symptoms of sleep disorders. Most of the residents mentioned that training is required to treat sleep disorders and suggested lifestyle modifications at the individual level and sleep clinics at PHCC. This study's results prompt administrators to send them to sleep medicine training and promote sleep medicine clinics at PHCC, the first level of care, to address productivity issues and decrease the load on tertiary care centers.

## Figures and Tables

**Figure 3:**
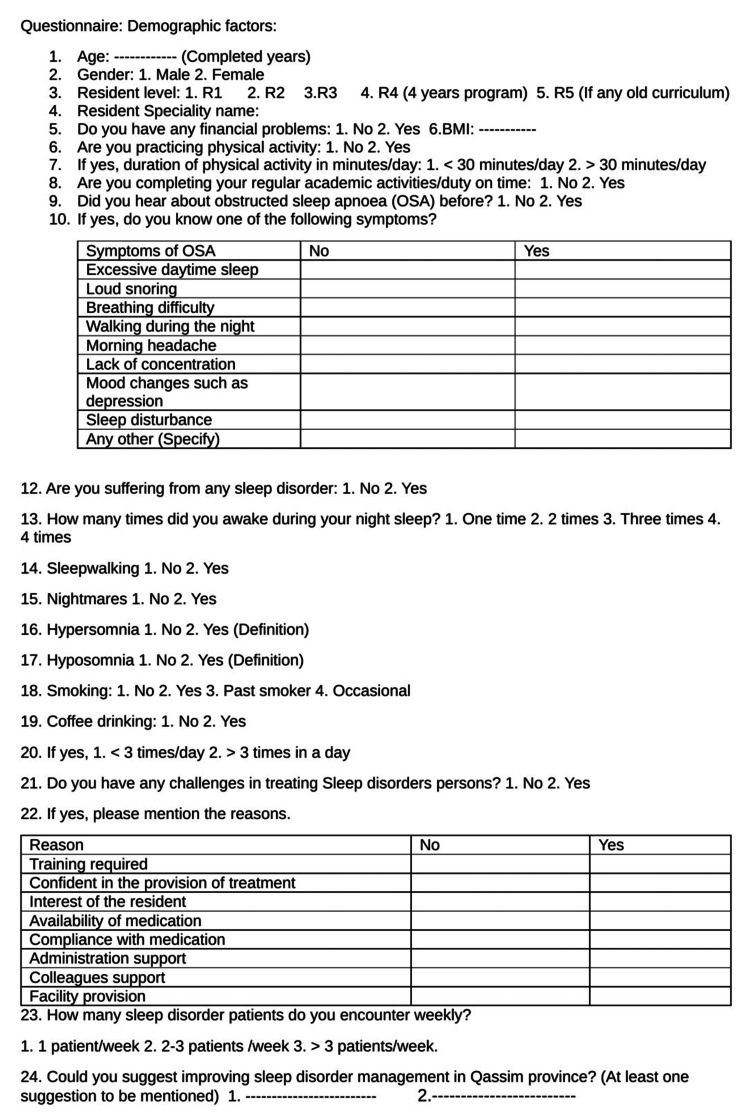
Questionnaire. BMI: body mass index

## Appendices

Figure 3 shows the questionnaire used in this study.
